# Where Next for Genetics and Genomics?

**DOI:** 10.1371/journal.pbio.1002216

**Published:** 2015-07-30

**Authors:** Chris Tyler-Smith, Huanming Yang, Laura F. Landweber, Ian Dunham, Bartha M. Knoppers, Peter Donnelly, Elaine R. Mardis, Michael Snyder, Gil McVean

**Affiliations:** 1 The Wellcome Trust Sanger Institute, Wellcome Trust Genome Campus, Hinxton, Cambridgeshire, United Kingdom; 2 BGI-Shenzen, Shenzen, China; 3 James D Watson Institute of Genome Science, Hangzhou, China; 4 Department of Ecology and Evolutionary Biology, Princeton University, Princeton, New Jersey, United States of America; 5 European Molecular Biology Laboratory, European Bioinformatics Institute (EMBL-EBI), Wellcome Trust Genome Campus, Hinxton, Cambridge, United Kingdom; 6 Centre of Genomics and Policy, McGill University, Montreal, Quebec, Canada; 7 The Wellcome Trust Centre for Human Genetics, University of Oxford, Oxford, United Kingdom; 8 Department of Statistics, University of Oxford, Oxford, United Kingdom; 9 McDonnell Genome Institute, School of Medicine, Washington University, St. Louis, Missouri, United States of America; 10 Department of Genetics, Stanford University, Stanford, California, United States of America

## Abstract

The last few decades have utterly transformed genetics and genomics, but what might the next ten years bring? *PLOS Biology* asked eight leaders spanning a range of related areas to give us their predictions. Without exception, the predictions are for more data on a massive scale and of more diverse types. All are optimistic and predict enormous positive impact on scientific understanding, while a recurring theme is the benefit of such data for the transformation and personalization of medicine. Several also point out that the biggest changes will very likely be those that we don’t foresee, even now.

## Abstract 2

The last few decades have utterly transformed genetics and genomics, but what might the next ten years bring? In this Perspective, eight leaders, spanning a range of related areas, give us their predictions.

This Perspective is part of the "Where Next?" Series.

## Introduction

### Chris Tyler-Smith

**Fig 1:**
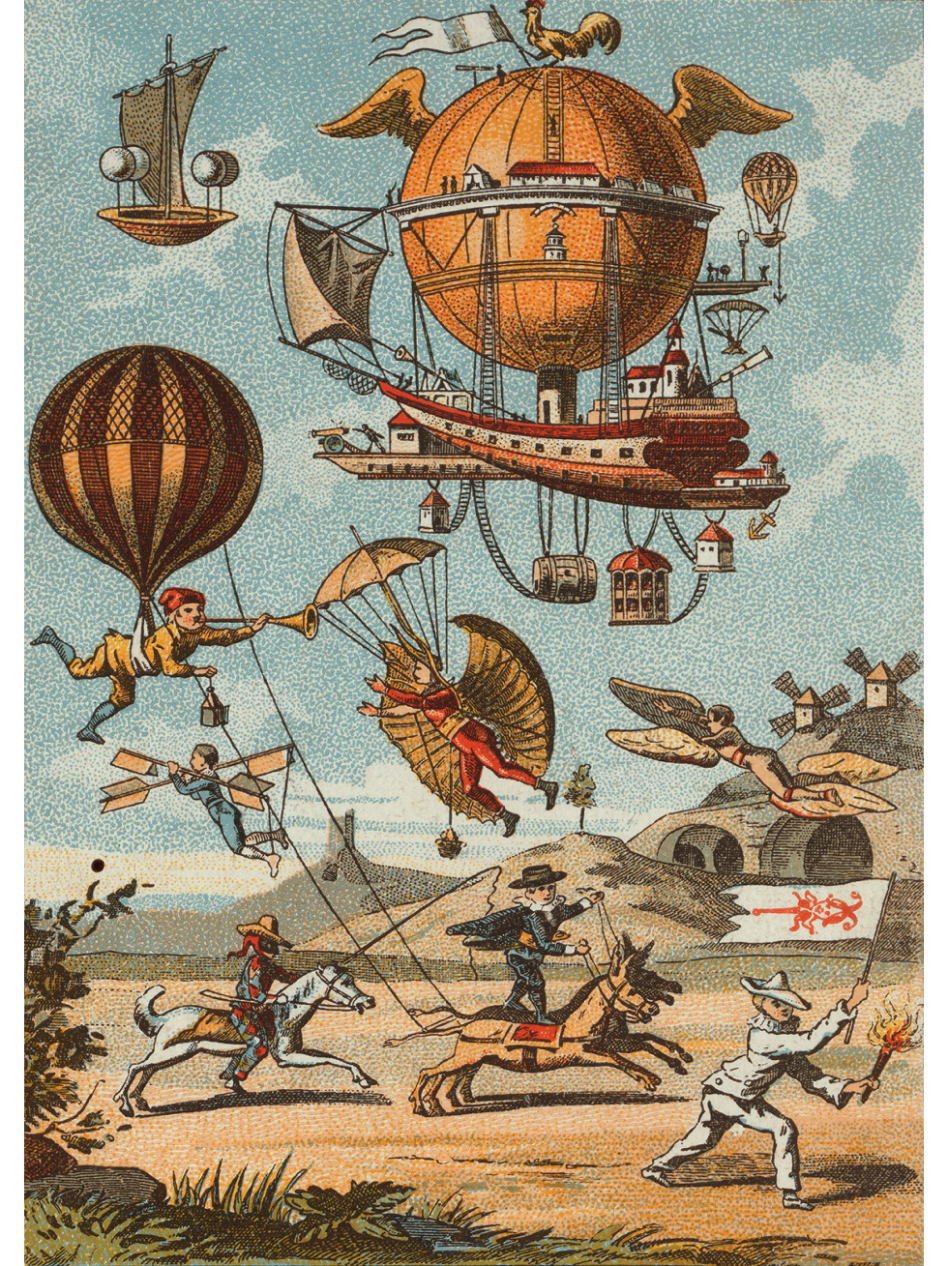
Predicting the future can be challenging. Romanet & cie, Paris; Collection 476, 2^e^ série, N°. 2—“Les utopies de la navigation aérienne au siècle dernier.” *Image credit*: *Public domain*, *from Wikimedia Commons*.

Predictions about the future of a rapidly developing field are usually doomed to be overtaken by events or proved wrong (Fig 1) but are nonetheless irresistible to both authors and readers. It is difficult to find a parallel for the developments in genomic technology over the last decade, exemplified by the halving of sequencing costs each six months for much of this period. Where will this lead?

A dead-safe bet is that we will have more sequences and indeed more data from all the omics technologies. A pretty-safe second bet is that our basic scientific understanding will benefit and that the implications for animal and plant breeding and—most of all—medicine, will be explored and lead to practical advances. We are given several examples of these possible directions. But how will society view such developments? There are no dystopian visions here, no predictions of an emerging antigenomics movement, but instead optimism that a fuller understanding of the contingency of the health and disease states will lead to destigmatization. Perhaps more surprisingly, there are no predictions of data overload or the dire consequences of analyzing such ever-increasing datasets using computers whose costs halve more slowly, perhaps only every 18 months. Somehow, we assume, we will deal with the data. We must hope so.

Enjoy these prognostications from our current leaders. Just bear in mind that the future will almost certainly be stranger and, consequently, even more exciting.

## The Diverse Applications of Genomics

### Huanming Yang

The Human Genome Project (HGP), the first endeavor and practice of genomics in its real sense, will further demonstrate its significance for reshaping life sciences and medicine by making life digital as a part of the "big data" era of the world. It is not difficult to predict the following in a few years:

A digitalized "Tree of Life" will be constructed by sequencing one or a few individuals of each of most, if not all, of the species identified on earth, which will change our views of the whole of life.

An open-source/free-access set of genome sequences of germ plasm for breeding plants and animals will be available by sequencing most, if not all, domestic cultivars and/or wild strains of these species to link the genomes and phenotypic traits, which will dramatically change the ways and efficiencies of breeding.

An integrated omics knowledge about humans will be generated by sequencing the whole genomes of at least one million individuals from most, if not all, ethnic groups, including both "normal" people and those suffering from various well-diagnosed diseases, which will lay the foundation of medicine and health care for the rest of the century: noninvasive prenatal testing as well as preimplantation testing will be applied to chromosomal-numerical-abnormal diseases and some monogenic diseases. Personalized protocols will serve patients with cancers and other complex diseases. Metagenomics will begin to enter the clinical field for many metabolic diseases. More importantly, the culture of collaboration cultivated by the HGP will spread. A balance between the principles of free access to genome sequencing data and proper protection of privacy, intellectual property rights, and society will be built. The mutual understanding of the scientific community and the public will be further harmonized.

The future is brilliant and is now.

## Cracking Open Nature

### Laura F. Landweber

The study of genome evolution is once again entering a new era, this time ushered in by long-read sequencing platforms. With several thousand base pairs per read, these are considerably longer than the best Sanger reads delivered in their prime, in contrast to the tiny reads that have dominated genome sequencing over the past few years. The new types of data permit a close examination of genome architectures never dreamt of before and offer surprisingly high resolution of even repetitive regions—once considered the “dark matter” of the genome. A much more complete view of genomes, chromosomes, and their evolutionary history will become feasible over the next decade. Protists, in particular, display a vast range of genome architectures, many notoriously replete with repeated regions, emphasizing the need for long-read sequencing technologies to infer the correct physical structure.

Protists often employ surprisingly sophisticated variations on DNA or RNA processing for genome editing. Their renegade pathways rewrite genomic information—eroding traditional notions of a gene and permitting them to tolerate extreme genome architectures and genetic systems [1]. For example, the ciliate *Oxytricha* supports two divergent architectures in two nuclei whose genomes differ radically, both from each other and from most other known forms of life. The macronucleus contains thousands of minimalist chromosomes that typically carry only a single gene each [2], while the micronucleus maintains a dispersed archive of nearly a quarter-million tiny DNA pieces—the nanochromosome precursors—on long, repeat-rich chromosomes [3]. Yet, *Oxytricha* represents just one evolutionary lineage that illustrates nature’s capriciousness regarding the storage and processing of genetic information. The next decade promises to reveal a bounty of information about the true range of life on our planet at its most fundamental level of genome diversity and genetic organization. I keenly look forward to the surprises, some with the potential to overturn textbook concepts of molecular biology once again.

## Functional Genome Annotation and In Silico Biology

### Ian Dunham

Genome sequencing projects have catalyzed huge steps toward identifying the biochemical entities that regulate and enact cellular differentiation and those that may become aberrant in disease. However, we are still at the beginning of understanding the wealth of different data types annotating the genome and how the many molecules in the parts list interact with each other to give function. In many ways, our understanding of genome-wide data is still influenced by the classical reductionist examples from early molecular biology and the idea that molecule X “does” function Y. Humans like to have stories to pin their understanding on, but these stories are often only the strongest effect and reflect a simplistic view of the molecular processes in the complex milieu of individual cells. The challenge then for the next decade is to develop methods of integration that seamlessly connect genome-wide data from the genetics of genome-wide association study (GWAS) and large-scale sequencing through chromatin immunoprecipitation sequencing (ChIP-seq) and RNA sequencing (RNA-seq) to tissue- and cell-specific behaviors in health and disease, allowing the effects of perturbation to be explored. This might be at the level of databases and sophisticated visual interfaces that allow smooth data exploration across the levels of resolution from a single cell through to the whole organism, with access to perturbed data from gene knockouts or disease, or it might be more complex modelling of the molecular relationships from the data, enabling predictions of effects of change through causal reasoning. These developments would really bring biology in silico.

## Genetics, Bioethics, and Equivalency of Risk

### Bartha Knoppers

Bioethics can offer no crystal ball predictions, dire or otherwise, for genetic-genomic futures. There are, however, Delphic signals of an emerging dynamic, integrative, and global approach to bioethics. These more humble signals stand in contrast to the reactive, overly cautious pronouncements characterizing bioethics over the past 25 years. Level-headed and proportional assessments are what bioethics needs. Consider this emerging approach in bioethics. It is dynamic as it moves toward building an Ethical, Legal, and Social Issues (ELSI) 2.0 initiative [4] to provide new tools for the policy challenges created by international research consortia and their transjurisdictional sharing of data and samples. It is integrative in recognizing that theoretical frameworks alone are not sufficient to build the policies necessary for influencing practice or to understand patient and research participants’ interests or behaviors. Hence, the need for empirical and qualitative data, as well as greater community engagement and interdisciplinary networking. Bioethics is also increasingly global and is focusing on issues of governance, accountability, and transparency.

Thus, as next-generation sequencing begins to break down the barriers between research and the clinic, as genomic and clinical data are responsibly integrated in the “cloud” to look for patterns of health and disease [5], and as rare-disease patients spurn the paternalistic ethics and laws that constrain international tissue and data sharing [6], bioethics must also move beyond the individual, beyond the specter of Nuremberg, and beyond genetic exceptionalism, so as to “renormalize” the individual. Indeed, by understanding the risk probabilities of resistance and susceptibility, by constructing infrastructure science such as biobanks as a basis for discovery science [7], and by stratifying risk to enable targeted health promotion and prevention, we may well ensure the sustainability of universal health care systems. Such stratification into subpopulations of risk or resistance may also serve to break down traditional classifications of age, gender, and disease as individuals are regrouped by probabilities into new health or disease communities. Is it utopian, then, to believe that today’s deluge of genetic/genomic information may thereby destigmatize individuals and democratize society by revealing equivalency of risk and equality in risk? Here, no crystal ball is needed.

## Complex Genetics: Anticipating a Billion Human Genome Sequences

### Peter Donnelly

Genomics has already had a major impact on our understanding of genetics. Although its likely short-term impact was overhyped, the HGP was definitely a turning point, followed by the availability of reference genomes for model systems and many other organisms. More recently, the ability to genotype millions of single nucleotide polymorphisms (SNPs) in thousands of individuals has transformed our knowledge of DNA sequence variants associated with common complex human diseases.

But it feels like we are now at another turning point. The cost of high-coverage whole human genomes has plummeted to the point at which this information is starting to be collected as part of clinical medical care. In the United Kingdom, for example, the National Health Service plans to sequence 100,000 patients by 2017. With appropriate consent, anonymization, and control, the sequence information will be linked to patients’ electronic medical records and made available to researchers. Genomic data linked to extensive phenotype and health information will be collected on millions of individuals within a few years, from clinical medicine and also research-driven biobanks. Within 15 years, there may be one billion humans whose genomes have been sequenced, in many cases with links to electronic health data.

In this future, complex genetic questions can be addressed empirically in silico. For example, we could learn about the health consequences of a mutation by collecting together clinical data on individuals carrying the mutation or about possible safety issues for a drug targeting a specific gene by studying individuals who carry genetic variants whose effects mimic, or are similar to, the proposed drug. There will be formidable challenges concerning both the access and the analysis of these vast, disconnected, and heterogeneous databases, but the rewards will be huge. The ability to edit genomes and measure consequences at scale, in cellular and animal systems, will complement this data explosion and allow novel findings to be validated experimentally.

## Miniaturized Genomic Monitoring

### Elaine Mardis

During my 30-year career in genomics, there have been remarkable advances in sequencing and associated technologies. Over the next decade, breakthroughs will no doubt continue, and with increasing impact on biomedical research and translational medicine. I am personally excited by the use of genomic technology for monitoring health. We have already multiple illustrations of this paradigm; for example, noninvasive prenatal testing is becoming part of the standard of maternity care, and patients on clinical trials of cancer therapies are receiving blood-based monitoring to determine if they are responding or experiencing progressive disease. So, where might this current trend lead us?

Cominiaturization of sequencing technology and of computational hardware will make it possible over the next ten years to use miniaturized personal monitoring devices to periodically or continually assay blood, urine, sputum, or other bodily fluids, automatically identifying genomic markers of disease and health. Although we currently use multistep benchtop sequencing technology to chronicle the progression or response of cancers to therapies by monitoring circulating tumor cells or free DNA in blood (“liquid” biopsy), over the next decade a miniaturized automated assay device could perform monitoring during and after cancer therapy. Proper testing could result in the use of such devices to predict the onset of cancer. While we presently react to medicate a fully developed viral or bacterial infection, daily monitoring would tell us about an emerging infection status. This information might keep us from going into work at our most infectious stage, thereby permitting early treatment to minimize symptoms while limiting the impact on coworkers. Indeed, with the correct analytics, the device would also tell us the best medication to take. For pregnancy, early detection of a new pregnancy could provide critical information concerning lifestyle alterations such as increased folic acid intake or smoking and alcohol cessation. Indeed, the future equivalent of the 1960s Star Trek tricorder would allow people to independently monitor their health on a daily basis, ensuring a proactive, rather than reactive, approach to health.

## Personalized Genomics: Towards a Proactive Model

### Michael Snyder

As the cost of high-throughput DNA sequencing continues to plummet and our ability to generate robust, clinically relevant information from genomics and other omics analyses increases, the impact on human health will be profound. Integrated omics information will be useful for elucidating mechanisms underlying aging, the unique properties of stem cells, and other important scientific questions related to human health. Genome sequencing (or targeted versions thereof) is already being used to tailor treatments for certain patients with cancer and other serious diseases, and these applications will likely become routine. Ultimately, in cancer patients, transcriptomes combined with genome information will be important in designing therapies. There will be a continuing evolution in how we think about and manage common complex diseases, such as type 2 diabetes, asthma, and autism, as patient subgroups with unique molecular signatures are defined accordingly and their specific treatment responses are categorized. Both individual molecular signatures as well as biological pathway information will become crucial for understanding and treating complex diseases. Targeted protein and metabolite analyses will become more routine for catching early disease states. Using sensors of biofluids (urine and blood drops) for routine monitoring of key molecules (nucleic acids, proteins, and metabolites) discovered through omics will become commonplace. Accompanying these developments will be an increase in the efficiency of clinical development, as omics information is used to guide how drugs are tested and in which patient subgroups.

Ideally, genomics will help move health management from a largely reactive model, in which diseases are treated after symptoms arise, into a more proactive model in which people obtain and use their genetic and epigenetic (e.g., DNA methylome and chromatin state) information to lower their risk of disease development by making individualized lifestyle changes and other interventions. In the long run, it likely will become common to obtain genomic information before birth, from the circulating fetal DNA in the mother’s blood.

Finally, the most significant development in personalized genomics in the next 10–15 years will probably be something we cannot anticipate based on current scientific knowledge. The power of high-throughput omics approaches to identify previously unrecognized patterns and trends, and drive novel hypothesis generation, could ultimately lead to research that fundamentally changes how we understand basic biological processes and human disease.

## Population Genetics: More Traits, More Populations, and More Species

### Gil McVean

The study of genetic variation has, over the last decade, been turned from a polite discipline focused on the finer points of evolutionary modelling to a fast, exhilarating, and sometimes messy hunt for gems hiding within the mines of genome-wide, population-scale datasets, most of which have been from humans. The coming years will only see the data rush grow: bigger samples, new species, extinct species, data linked to phenotype, temporal data, and so on. What, in this great whirlwind, am I most excited by?

Data are at their most fun when they bring to light things you would never have imagined. In humans, we’ve seen recombination hotspots that shift location, local selection for variants that influence traits from height to earwax type, polymorphisms that are millions of years old, unexpected paths taken on the long road out of Africa, and the genetic ghosts of archaic humans living on in the modern world, to name but a (subjective) few.

However, data can be at their most powerful when they are used to revisit some of those big questions in evolution that never went away. How does adaptation actually work? Is it similar in all species, or are there characteristics of a population that affect the architecture of a selection response? What is a species anyway, and how do they form? Do the fundamental parameters of genetic change—mutation and recombination—evolve? And if so, how and why? These are all questions that are best tackled by a comparative approach—multiple traits, multiple populations, and multiple species. So what I’m looking forward to is, of course, more data. But this time I don’t just want it from one (albeit mildly interesting) species. I want it from all of them.

## Abbreviations:

ChIP-seq: chromatin immunoprecipitation sequencing
ELSI: Ethical, Legal, and Social Issues
GWAS: genome-wide association study
HGP: Human Genome Project
RNA-seq: RNA sequencing
SNP: single nucleotide polymorphism
